# Two-Year Follow Up of Olanzapine Long-Acting Injection: A Retrospective Observational Study at the Maudsley Hospital Olanzapine Clinic.

**DOI:** 10.1192/bjo.2026.11613

**Published:** 2026-06-30

**Authors:** An Nakamura, Eunji Hong, Kalliopi Vallianatou, Ivana Clark, David Taylor

**Affiliations:** 1 Kings College London, Faculty of Life Sciences and Medicine, London, United Kingdom.; 2 South London and Maudsley NHS Foundation Trust, London, United Kingdom

## Abstract

**Aims:**

Olanzapine long-acting injection (OLAI) is prescribed as maintenance antipsychotic treatment within small established clinics in Southwark and Lambeth. Despite the association between long-acting antipsychotics use and reductions in the number of relapses and hospital admissions, and oral olanzapine being the most commonly prescribed antipsychotic, OLAI is the least commonly prescribed LAI. This is due to staff, space and funding resources needed for post-injection monitoring, a requirement unique to OLAI. In order to provide equitable access to evidence-based treatments like OLAI within the Trust, provision of OLAI must be expanded. This observational, non-interventional, retrospective study aims to measure a range of relevant outcomes in order to demonstrate the achievement of good treatment value.

**Methods:**

25 patients were initiated on OLAI between January 2020 and January 2022 and were followed up for 2 years. Our main outcome measures are: continuation rates of OLAI over the 2 year follow-up period, and reasons for stopping, relapse rates (number of bed days and hospital admissions) 2 years before and after starting OLAI, adherence to physical health and side-effect monitoring, and physical health outcomes. A retrospective review of general clinical patient notes and OLAI clinic notes was conducted.

**Results:**

Out of the 21 patients included in analysis, the 2 year continuation rate was 42.9% (n=9). Within these 9 patients, there was a notable reduction in the mean number of bed days, and zero admissions during the 2 year follow-up period. The most common reasons for discontinuation were adverse effects, main one being weight gain, followed by post-injection monitoring. Physical health monitoring during the 2 year follow-up period was inconsistent, this may partly be due to the Covid-19 pandemic. Weight was most consistently measured, however blood test measures were done less frequently, and declined over time. For the 9 patients who were followed up for 2 years, weight increased by a mean of 7.42kg.

**Conclusion:**

Whilst OLAI appears to have a very positive effect on the rate of relapse, especially with the reduction of admissions to zero, adverse effects and post-injection monitoring requirements may discourage patients from continuing. Next steps include embedding processes to ensure all physical health parameters are monitored, consider preventative measures against common adverse effects, and activities to improve acceptability of post-injection monitoring. By doing so, we can continue to promote patient safety as OLAI provision expands within the Trust. Co-authored with Dr Juliet Hurn, Consultant Psychiatrist at South London and Maudsley NHS Foundation Trust.

